# The aluminoarsenate Na_1.67_K_1.33_Al_3_(AsO_4_)_4_

**DOI:** 10.1107/S1600536813033850

**Published:** 2013-12-24

**Authors:** Mohamad Alem Bouhassine, Habib Boughzala

**Affiliations:** a Laboratoire des Materiaux et de Cristallochimie, Faculte des Sciences de Tunis, Tunisia

## Abstract

The title compound, sodium potassium trialuminium tetra­kis­(orthoarsenate), was prepared by solid-state reactions. The anionic framework consists of corrugated layers parallel to (010) and is made up of corner-sharing AlO_6_ octa­hedra (site symmetries .2. and 2/*m*..) that are connected to isolated AsO_4_ tetra­hedra (site symmetries .2. and *m*..) through edge- and corner-sharing. The alkali cations are occupationally disordered. The two K^+^ cations [site symmetries .2. and *m*..; occupancies 0.314 (7) and 0.035 (12)] are situated in the inter­layer space, whereas the smaller Na^+^ cations [both with site symmetry *m*..; occupancies = 0.725 (14) and 0.112 (14)] are located in the cavities of the anionic framework. The K^+^ cations are surrounded by six and seven O atoms, the Na^+^ cations by seven and nine O atoms. The resulting coordination polyhedra of the two types of cations are highly distorted.

## Related literature   

For further information on this structure type, see: Friaa *et al.* (2003 ▶); Haj Abdallah & Haddad (2012 ▶). For background to the bond-valence method, see: Brown (2002 ▶).

## Experimental   

### 

#### Crystal data  

Na_1.67_K_1.33_Al_3_(AsO_4_)_4_
*M* *_r_* = 726.95
Orthorhombic, 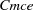

*a* = 10.493 (1) Å
*b* = 20.395 (4) Å
*c* = 6.335 (4) Å
*V* = 1355.7 (9) Å^3^
*Z* = 4
Mo *K*α radiation
μ = 10.53 mm^−1^
*T* = 293 K
0.40 × 0.10 × 0.07 mm

#### Data collection  

Enraf–Nonius CAD-4 diffractometer
Absorption correction: ψ scan (North *et al.*, 1968 ▶) *T*_min_ = 0.130, *T*_max_ = 0.332
1723 measured reflections
780 independent reflections
717 reflections with *I* > 2σ(*I*)
*R*_int_ = 0.062
2 standard reflections every 120 min intensity decay: 1.1%

#### Refinement  

*R*[*F* ^2^ > 2σ(*F* ^2^)] = 0.038
*wR*(*F* ^2^) = 0.112
*S* = 1.15
780 reflections
84 parameters
1 restraint
Δρ_max_ = 2.38 e Å^−3^
Δρ_min_ = −1.33 e Å^−3^

### 

Data collection: *CAD-4 EXPRESS* (Enraf–Nonius, 1994 ▶); cell refinement: *CAD-4 EXPRESS*; data reduction: *XCAD4* (Harms & Wocadlo, 1995 ▶); program(s) used to solve structure: *SHELXS97* (Sheldrick, 2008 ▶); program(s) used to refine structure: *SHELXL97* (Sheldrick, 2008 ▶); molecular graphics: *DIAMOND* (Brandenburg, 2006 ▶); software used to prepare material for publication: *publCIF* (Westrip, 2010 ▶).

## Supplementary Material

Crystal structure: contains datablock(s) I, New_Global_Publ_Block. DOI: 10.1107/S1600536813033850/wm2786sup1.cif

Structure factors: contains datablock(s) I. DOI: 10.1107/S1600536813033850/wm2786Isup2.hkl

Additional supporting information:  crystallographic information; 3D view; checkCIF report

## Figures and Tables

**Table 1:** Selected bond lengths (Å)

As1—O1	1.642 (5)
As1—O2^i^	1.678 (3)
As1—O2	1.678 (3)
As1—O3	1.694 (4)
As2—O4^ii^	1.648 (3)
As2—O4	1.648 (3)
As2—O5	1.729 (3)
As2—O5^ii^	1.729 (3)
Al1—O3	1.827 (5)
Al1—O3^iii^	1.827 (5)
Al1—O5^iii^	1.947 (3)
Al1—O5^iv^	1.947 (3)
Al1—O5^i^	1.947 (3)
Al1—O5	1.947 (3)
Al2—O2^ii^	1.817 (3)
Al2—O2	1.817 (3)
Al2—O4^v^	1.924 (3)
Al2—O4^vi^	1.924 (3)
Al2—O5^ii^	1.991 (3)
Al2—O5	1.991 (3)

Symmetry codes: (i) 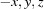; (ii) 
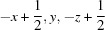
; (iii) 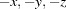; (iv) 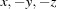; (v) 
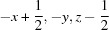
; (vi) 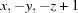.

**Fig. 1.**
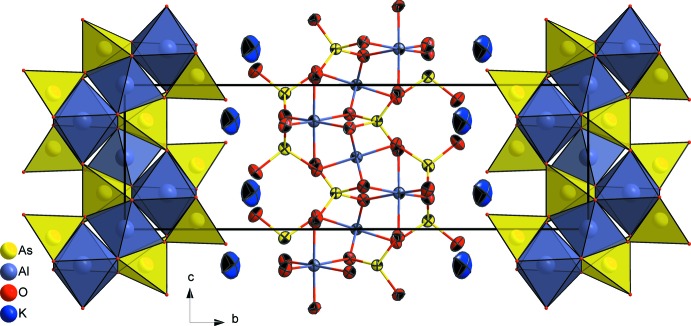
Projection of the Na1.67K1.33Al3(AsO4)4 structure along [100], showing the K1+ and K2+ cations in the corrugated interlayer space. Displacement ellipsoids are drawn at the 90% probability level.

**Fig. 2.**
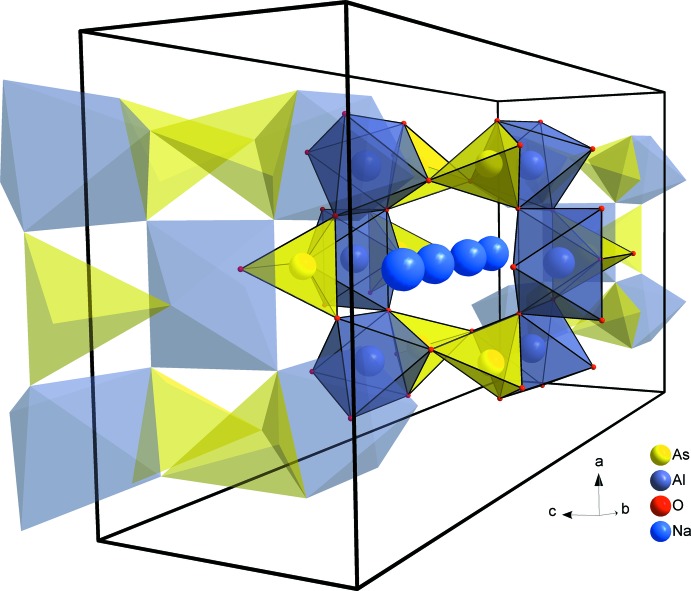
Cavities limited by the anionic framework hosting Na+ cations in the Na1.67K1.33Al3(AsO4)4 structure.

## supplementary crystallographic information

### 1. Comment

Na_1.67_K_1.33_Al_3_(AsO_4_)_4_ is isostructural with K_3_Cr_3_(AsO_4_)_4_ (Friaa *et al.* (2003)) and belongs to the *A*^I^*M*^III^(*X*O_4_)_4_ family of compounds (*A*^I^ = alkali metal, *M*^III^ = Al, Cr, Fe,.. *X* = As, P) . Its structure consists of AlO_6_ octahedra and AsO_4_ tetrahedra sharing corners and edges to form a two-dimensional framework (Fig.1) that consists of corrugated layers extending parallel to (010). Each layer lies on a *c*-glide plane perpendicular to the *b* axis at *y* = 0 and *y* = 1/2. In the asymmetric unit two AlO_6_ octahedra are linked to an As1O_4_ tetrahedron by sharing corners and to an As2O_4_ tetrahedron by sharing edges. The alkali cations are occupationally disordered with occupancies of 0.725 (14), 0.112 (14), 0.314 (7) and 0.035 (12) for Na1, Na2, K1 and K2, leading to the chemical formula Na_1.67_K_1.33_Al_3_(AsO_4_)_4_. Smaller than K^+^, the seven- and nine-coordinated Na1^+^ and Na2^+^ (r_Na+_ = 1.02 Å), are located in cavities surrounded by the anionic framework (Fig. 2). On the other hand, the K1^+^ and K2^+^ cations (r_K+_ = 1.38 Å) are surrounded, respectively, by seven and six O atoms and are located in larger sites in the corrugated interlayer space. Using the bond valence method (Brown, 2002), the calculated bond-valence sum values (in valence units) of 5.17, 4.98, 3.04, 3.03, 0.86, 1.11, 0.92, 0.86, respectively, for As1, As2, Al1, Al2, Na1, Na2, K1 and K2 are in good agreement with the expected oxidation states.

A similar distribution of cations was observed in K_1.8_Sr_0.6_Al_3_(AsO_4_)_4_ (Haj Abdallah *et al.*, 2012). In fact, smaller than K^+^, the Sr^2+^ cations (r_Sr2+_ = 1.18 Å) are located in the anionic cavities, on the other hand, K^+^ occupy the interlayer space. The cationic distribution in these two structures make it reasonable to conclude that the interlayer space is reserved for bigger cations and the anionic cavities for smaller cations. To our knowledge, this is the first published structure with occupationally disordered alkali sites in the *A*^I^*M*^III^(*X*O_4_)_4_ family of compounds. Reports of some new members of this family will be published soon.

### 2. Experimental

Crystals of Na_1.67_K_1.33_Al_3_(AsO_4_)_4_ were obtained from a mixture of K_2_CO_3_, Al_2_O_3_ and NH_4_H_2_AsO_4_ in the stoichiometric molar ratio K/Al/As=2/3/4. The mixture was finely ground and heated in a porcelain crucible at 723 K for 4 h to eliminate volatile products. The temperature was held at 1173 K during 10 days until fusion was reached. The sample was slowly cooled in a speed of 5 K/ h to 923 K and finally quenched to room temperature. A long wash with boiling water liberated colorless crystals. The qualitative analysis by electron microscope probe of a selected crystal revealed the presence of the different elements in the compound composition. It is most likely that the incorporated sodium stems from the crucible.

### 3. Refinement

Except for K2 and Na2, both with a very low occupation, all atoms were refined with anisotropic displacement parameters. Constraints were applied to the Na^+^ and K^+^ cation occupation rates to achieve electro-neutrality. The highest and lowest values of the electron densities occurare located 0.97 Å and 0.80 Å, respectively, from As2.

### Crystal data

**Table:**

Na_1.67_K_1.33_Al_3_(AsO_4_)_4_	*F*(000) = 1370.4
*M**_r_* = 726.95	*D*_x_ = 3.563 Mg m^−^^3^
Orthorhombic, *C**m**c**e*	Mo *K*α radiation, λ = 0.71073 Å
Hall symbol: -C 2bc 2	Cell parameters from 25 reflections
*a* = 10.493 (1) Å	θ = 3.8–27°
*b* = 20.395 (4) Å	µ = 10.53 mm^−^^1^
*c* = 6.335 (4) Å	*T* = 293 K
*V* = 1355.7 (9) Å^3^	Plate, colourless
*Z* = 4	0.40 × 0.10 × 0.07 mm

### Data collection

**Table:**

Enraf–Nonius CAD-4 diffractometer	717 reflections with *I* > 2σ(*I*)
Radiation source: fine-focus sealed tube	*R*_int_ = 0.062
Graphite monochromator	θ_max_ = 27.0°, θ_min_ = 3.8°
ω/2θ scans	*h* = −13→1
Absorption correction: ψ scan (North *et al.*, 1968)	*k* = −26→1
*T*_min_ = 0.130, *T*_max_ = 0.332	*l* = −8→8
1723 measured reflections	2 standard reflections every 120 min
780 independent reflections	intensity decay: 1.1%

### Refinement

**Table:**

Refinement on *F*^2^	1 restraint
Least-squares matrix: full	Primary atom site location: structure-invariant direct methods
*R*[*F*^2^ > 2σ(*F*^2^)] = 0.038	Secondary atom site location: difference Fourier map
*wR*(*F*^2^) = 0.112	*w* = 1/[σ^2^(*F*_o_^2^) + (0.0727*P*)^2^ + 0.5867*P*] where *P* = (*F*_o_^2^ + 2*F*_c_^2^)/3
*S* = 1.15	(Δ/σ)_max_ < 0.001
780 reflections	Δρ_max_ = 2.38 e Å^−^^3^
84 parameters	Δρ_min_ = −1.33 e Å^−^^3^

### Special details

**Table:**

Geometry. All e.s.d.'s (except the e.s.d. in the dihedral angle between two l.s. planes) are estimated using the full covariance matrix. The cell e.s.d.'s are taken into account individually in the estimation of e.s.d.'s in distances, angles and torsion angles; correlations between e.s.d.'s in cell parameters are only used when they are defined by crystal symmetry. An approximate (isotropic) treatment of cell e.s.d.'s is used for estimating e.s.d.'s involving l.s. planes.
Refinement. Refinement of *F*^2^ against ALL reflections. The weighted *R*-factor *wR* and goodness of fit *S* are based on *F*^2^, conventional *R*-factors *R* are based on *F*, with *F* set to zero for negative *F*^2^. The threshold expression of *F*^2^ > σ(*F*^2^) is used only for calculating *R*-factors(gt) *etc*. and is not relevant to the choice of reflections for refinement. *R*-factors based on *F*^2^ are statistically about twice as large as those based on *F*, and *R*- factors based on ALL data will be even larger.

### Fractional atomic coordinates and isotropic or equivalent isotropic displacement parameters (Å^2^)

**Table:**

	*x*	*y*	*z*	*U*_iso_*/*U*_eq_	Occ. (<1)
As1	0.0000	0.15486 (3)	0.05909 (10)	0.0075 (3)	
As2	0.2500	−0.04448 (3)	0.2500	0.0070 (3)	
Al1	0.0000	0.0000	0.0000	0.0063 (5)	
Al2	0.2500	0.09187 (10)	0.2500	0.0073 (4)	
O1	0.0000	0.2179 (3)	−0.1020 (8)	0.0163 (10)	
O2	0.1263 (3)	0.15363 (15)	0.2215 (5)	0.0110 (7)	
O3	0.0000	0.0854 (2)	−0.0873 (7)	0.0095 (9)	
O4	0.2105 (3)	−0.09062 (16)	0.4534 (4)	0.0102 (6)	
O5	0.1364 (3)	0.01511 (13)	0.2027 (5)	0.0086 (6)	
Na1	0.0000	0.1628 (3)	0.5513 (7)	0.0350 (18)	0.725 (14)
Na2	0.0000	0.0635 (17)	0.520 (5)	0.042 (12)*	0.112 (14)
K1	0.2125 (6)	0.2737 (3)	0.1075 (10)	0.048 (2)	0.314 (7)
K2	0.2500	0.271 (3)	0.2500	0.03 (2)*	0.035 (12)

### Atomic displacement parameters (Å^2^)

**Table:**

	*U*^11^	*U*^22^	*U*^33^	*U*^12^	*U*^13^	*U*^23^
As1	0.0016 (4)	0.0103 (4)	0.0108 (4)	0.000	0.000	0.0013 (2)
As2	0.0039 (4)	0.0086 (4)	0.0084 (4)	0.000	−0.0005 (2)	0.000
Al1	0.0011 (10)	0.0090 (12)	0.0089 (11)	0.000	0.000	0.0003 (10)
Al2	0.0028 (9)	0.0093 (9)	0.0099 (9)	0.000	−0.0001 (6)	0.000
O1	0.020 (3)	0.013 (2)	0.016 (2)	0.000	0.000	0.006 (2)
O2	0.0045 (15)	0.0124 (15)	0.0161 (15)	0.0010 (11)	−0.0040 (13)	−0.0019 (11)
O3	0.006 (2)	0.009 (2)	0.0133 (19)	0.000	0.000	−0.0024 (17)
O4	0.0091 (15)	0.0116 (15)	0.0100 (13)	−0.0029 (12)	0.0009 (12)	0.0017 (11)
O5	0.0033 (14)	0.0105 (15)	0.0120 (13)	0.0023 (11)	−0.0018 (13)	−0.0007 (10)
Na1	0.037 (3)	0.047 (3)	0.020 (3)	0.000	0.000	−0.0033 (19)
K1	0.053 (3)	0.034 (3)	0.059 (4)	−0.029 (3)	0.013 (3)	0.003 (2)

### Geometric parameters (Å, º)

**Table:**

As1—O1	1.642 (5)	O4—Na2^xv^	2.284 (9)
As1—O2^i^	1.678 (3)	O4—Na1^xv^	2.655 (4)
As1—O2	1.678 (3)	O4—K1^xvi^	2.795 (6)
As1—O3	1.694 (4)	O4—K2^xvi^	3.13 (5)
As2—O4^ii^	1.648 (3)	O5—Na2	2.66 (3)
As2—O4	1.648 (3)	O5—Na2^xv^	2.77 (3)
As2—O5	1.729 (3)	Na1—Na2	2.03 (4)
As2—O5^ii^	1.729 (3)	Na1—O1^xvii^	2.467 (7)
As2—Al2	2.781 (2)	Na1—O2^i^	2.481 (5)
Al1—O3	1.827 (5)	Na1—K1^viii^	2.603 (8)
Al1—O3^iii^	1.827 (5)	Na1—K1^xviii^	2.603 (8)
Al1—O5^iii^	1.947 (3)	Na1—O1^xviii^	2.619 (8)
Al1—O5^iv^	1.947 (3)	Na1—O4^xv^	2.655 (4)
Al1—O5^i^	1.947 (3)	Na1—O4^vi^	2.655 (4)
Al1—O5	1.947 (3)	Na1—O3^xvii^	2.781 (7)
Al2—O2^ii^	1.817 (3)	Na1—K2^xix^	3.21 (2)
Al2—O2	1.817 (3)	Na2—O4^vi^	2.284 (9)
Al2—O4^v^	1.924 (3)	Na2—O4^xv^	2.284 (9)
Al2—O4^vi^	1.924 (3)	Na2—O3^xvii^	2.53 (3)
Al2—O5^ii^	1.991 (3)	Na2—Na2^xv^	2.60 (7)
Al2—O5	1.991 (3)	Na2—O5^i^	2.66 (3)
Al2—Na2	3.19 (2)	Na2—O5^vi^	2.77 (3)
Al2—Na2^vii^	3.19 (2)	Na2—O5^xv^	2.77 (3)
Al2—Na1^vii^	3.552 (3)	Na2—O2^i^	2.95 (3)
Al2—Na1	3.552 (3)	Na2—As2^xiv^	3.025 (17)
Al2—K1^viii^	3.579 (6)	K1—K2	0.986 (7)
O1—Na1^ix^	2.467 (7)	K1—K1^xi^	1.846 (12)
O1—Na1^x^	2.620 (8)	K1—K1^ii^	1.969 (14)
O1—K2^x^	2.795 (5)	K1—K2^xi^	2.47 (2)
O1—K2^xi^	2.795 (5)	K1—Na1^x^	2.603 (8)
O1—K1^i^	2.833 (7)	K1—O4^xx^	2.795 (6)
O1—K1	2.833 (7)	K1—O1^xviii^	2.896 (6)
O1—K1^x^	2.896 (6)	K1—O2^xii^	2.999 (7)
O1—K1^xii^	2.896 (6)	K1—O1^xi^	3.022 (6)
O1—K1^xi^	3.022 (6)	K1—O2^xi^	3.067 (6)
O1—K1^xiii^	3.022 (6)	K2—K1^ii^	0.986 (7)
O2—Na1	2.481 (5)	K2—K1^viii^	2.47 (2)
O2—K1	2.708 (6)	K2—K1^xi^	2.47 (2)
O2—K2	2.73 (5)	K2—O2^ii^	2.73 (5)
O2—Na2	2.95 (3)	K2—O1^xi^	2.795 (5)
O2—K1^viii^	2.999 (7)	K2—O1^xviii^	2.795 (5)
O2—K1^xi^	3.067 (6)	K2—O4^xx^	3.13 (5)
O2—K1^ii^	3.166 (7)	K2—O4^xxi^	3.13 (5)
O3—Na2^ix^	2.53 (3)	K2—Na1^x^	3.21 (2)
O3—Na1^ix^	2.781 (7)	K2—Na1^xix^	3.21 (2)
O4—Al2^xiv^	1.924 (3)		
			
O1—As1—O2^i^	113.13 (15)	O4^xv^—Na2—O2	109.3 (12)
O1—As1—O2	113.13 (15)	O3^xvii^—Na2—O2	121.4 (11)
O2^i^—As1—O2	104.3 (2)	Na2^xv^—Na2—O2	123.8 (17)
O1—As1—O3	108.4 (3)	O5^i^—Na2—O2	89.3 (10)
O2^i^—As1—O3	108.86 (14)	O5—Na2—O2	60.3 (6)
O2—As1—O3	108.86 (14)	O5^vi^—Na2—O2	122.25 (12)
O4^ii^—As2—O4	110.4 (2)	O5^xv^—Na2—O2	175.4 (8)
O4^ii^—As2—O5	116.04 (14)	O2^i^—Na2—O2	53.4 (6)
O4—As2—O5	111.32 (15)	Na1—Na2—As2^xiv^	94.7 (8)
O4^ii^—As2—O5^ii^	111.32 (15)	O4^vi^—Na2—As2^xiv^	32.5 (3)
O4—As2—O5^ii^	116.04 (14)	O4^xv^—Na2—As2^xiv^	146.6 (14)
O5—As2—O5^ii^	90.65 (19)	O3^xvii^—Na2—As2^xiv^	63.2 (6)
O4^ii^—As2—Al2	124.81 (11)	Na2^xv^—Na2—As2^xiv^	85.4 (10)
O4—As2—Al2	124.81 (11)	O5^i^—Na2—As2^xiv^	141.6 (11)
O5—As2—Al2	45.33 (10)	O5—Na2—As2^xiv^	81.4 (3)
O5^ii^—As2—Al2	45.33 (10)	O5^vi^—Na2—As2^xiv^	34.3 (3)
O3—Al1—O3^iii^	180.0	O5^xv^—Na2—As2^xiv^	93.9 (10)
O3—Al1—O5^iii^	87.19 (13)	O2^i^—Na2—As2^xiv^	141.0 (10)
O3^iii^—Al1—O5^iii^	92.81 (13)	O2—Na2—As2^xiv^	89.9 (4)
O3—Al1—O5^iv^	87.19 (13)	O2—K1—O4^xx^	158.4 (3)
O3^iii^—Al1—O5^iv^	92.81 (13)	K2—K1—O1	136 (2)
O5^iii^—Al1—O5^iv^	94.60 (18)	K1^xi^—K1—O1	77.3 (4)
O3—Al1—O5^i^	92.80 (13)	K1^ii^—K1—O1	138.1 (3)
O3^iii^—Al1—O5^i^	87.20 (13)	K2^xi^—K1—O1	63.1 (4)
O5^iii^—Al1—O5^i^	85.40 (18)	Na1^x^—K1—O1	57.43 (19)
O5^iv^—Al1—O5^i^	180.0	O2—K1—O1	59.97 (16)
O3—Al1—O5	92.80 (13)	O4^xx^—K1—O1	109.1 (3)
O3^iii^—Al1—O5	87.19 (13)	K2—K1—O1^xviii^	74.3 (4)
O5^iii^—Al1—O5	180.0	K1^xi^—K1—O1^xviii^	145.9 (4)
O5^iv^—Al1—O5	85.40 (18)	K1^ii^—K1—O1^xviii^	74.1 (3)
O5^i^—Al1—O5	94.60 (18)	K2^xi^—K1—O1^xviii^	136.6 (5)
O2^ii^—Al2—O2	92.3 (2)	Na1^x^—K1—O1^xviii^	53.00 (18)
O2^ii^—Al2—O4^v^	87.27 (15)	O2—K1—O1^xviii^	68.10 (17)
O2—Al2—O4^v^	93.78 (15)	O4^xx^—K1—O1^xviii^	91.3 (2)
O2^ii^—Al2—O4^vi^	93.78 (15)	O1—K1—O1^xviii^	73.48 (15)
O2—Al2—O4^vi^	87.27 (15)	K2—K1—O2^xii^	153 (3)
O4^v^—Al2—O4^vi^	178.5 (2)	K1^xi^—K1—O2^xii^	77.6 (4)
O2^ii^—Al2—O5^ii^	95.88 (12)	K1^ii^—K1—O2^xii^	150.20 (12)
O2—Al2—O5^ii^	171.00 (15)	K2^xi^—K1—O2^xii^	59.0 (10)
O4^v^—Al2—O5^ii^	90.44 (14)	Na1^x^—K1—O2^xii^	51.99 (17)
O4^vi^—Al2—O5^ii^	88.37 (14)	O2—K1—O2^xii^	124.3 (2)
O2^ii^—Al2—O5	171.00 (15)	O4^xx^—K1—O2^xii^	52.81 (14)
O2—Al2—O5	95.89 (12)	O1—K1—O2^xii^	65.11 (18)
O4^v^—Al2—O5	88.37 (14)	O1^xviii^—K1—O2^xii^	104.9 (3)
O4^vi^—Al2—O5	90.44 (14)	K2—K1—O1^xi^	67.3 (4)
O5^ii^—Al2—O5	76.27 (18)	K1^xi^—K1—O1^xi^	66.1 (4)
O2^ii^—Al2—As2	133.87 (11)	K1^ii^—K1—O1^xi^	67.1 (3)
O2—Al2—As2	133.87 (11)	K2^xi^—K1—O1^xi^	81.5 (2)
O4^v^—Al2—As2	89.24 (12)	Na1^x^—K1—O1^xi^	145.6 (2)
O4^vi^—Al2—As2	89.24 (12)	O2—K1—O1^xi^	112.8 (3)
O5^ii^—Al2—As2	38.14 (9)	O4^xx^—K1—O1^xi^	87.10 (17)
O5—Al2—As2	38.14 (9)	O1—K1—O1^xi^	143.4 (2)
As1—O2—Al2	129.43 (18)	O1^xviii^—K1—O1^xi^	140.6 (2)
As1—O3—Al1	129.2 (3)	O2^xii^—K1—O1^xi^	105.27 (19)
As2—O4—Al2^xiv^	135.8 (2)	K2—K1—O2^xi^	115.2 (17)
As2—O5—Al1	120.66 (15)	K1^xi^—K1—O2^xi^	61.0 (3)
As2—O5—Al2	96.54 (14)	K1^ii^—K1—O2^xi^	113.7 (4)
Al1—O5—Al2	131.59 (15)	K2^xi^—K1—O2^xi^	57.9 (10)
O2—Na1—O3^xvii^	130.6 (2)	Na1^x^—K1—O2^xi^	98.0 (2)
K1^viii^—Na1—O3^xvii^	99.79 (19)	O2—K1—O2^xi^	143.4 (2)
K1^xviii^—Na1—O3^xvii^	99.79 (19)	O4^xx^—K1—O2^xi^	55.33 (12)
O1^xviii^—Na1—O3^xvii^	146.4 (2)	O1—K1—O2^xi^	108.0 (2)
O4^xv^—Na1—O3^xvii^	72.21 (14)	O1^xviii^—K1—O2^xi^	145.9 (3)
O4^vi^—Na1—O3^xvii^	72.21 (14)	O2^xii^—K1—O2^xi^	51.17 (15)
Na2—Na1—As1	81.5 (10)	O1^xi^—K1—O2^xi^	54.13 (14)
O1^xvii^—Na1—As1	155.8 (3)	K1^ii^—K2—K1	174 (6)
O2^i^—Na1—As1	32.36 (9)	K1^ii^—K2—K1^viii^	40.9 (15)
O2—Na1—As1	32.36 (9)	K1—K2—K1^viii^	142 (2)
K1^viii^—Na1—As1	99.35 (18)	K1^ii^—K2—K1^xi^	142 (2)
K1^xviii^—Na1—As1	99.35 (18)	K1—K2—K1^xi^	40.9 (15)
O1^xviii^—Na1—As1	71.20 (16)	K1^viii^—K2—K1^xi^	137 (2)
O4^xv^—Na1—As1	87.71 (12)	K1^ii^—K2—O2	107 (3)
O4^vi^—Na1—As1	87.71 (12)	K1—K2—O2	78 (3)
O3^xvii^—Na1—As1	142.4 (2)	K1^viii^—K2—O2	70.1 (11)
Na2—Na1—K2^xix^	117.1 (10)	K1^xi^—K2—O2	72.0 (11)
O1^xvii^—Na1—K2^xix^	57.3 (3)	K1^ii^—K2—O2^ii^	78 (3)
O2^i^—Na1—K2^xix^	143.1 (5)	K1—K2—O2^ii^	107 (3)
O2—Na1—K2^xix^	85.70 (14)	K1^viii^—K2—O2^ii^	72.0 (11)
K1^viii^—Na1—K2^xix^	15.5 (4)	K1^xi^—K2—O2^ii^	70.1 (11)
K1^xviii^—Na1—K2^xix^	116.0 (8)	O2—K2—O2^ii^	57.3 (11)
O1^xviii^—Na1—K2^xix^	75.8 (9)	K1^ii^—K2—O1^xi^	85.9 (5)
O4^xv^—Na1—K2^xix^	156.7 (5)	K1—K2—O1^xi^	93.7 (5)
O4^vi^—Na1—K2^xix^	63.7 (8)	K1^viii^—K2—O1^xi^	119.1 (6)
O3^xvii^—Na1—K2^xix^	85.1 (6)	K1^xi^—K2—O1^xi^	64.7 (2)
As1—Na1—K2^xix^	114.45 (17)	O2—K2—O1^xi^	119.6 (16)
Na1—Na2—O4^vi^	75.6 (9)	O2^ii^—K2—O1^xi^	69.3 (6)
Na1—Na2—O4^xv^	75.6 (9)	K1^ii^—K2—O1^xviii^	93.7 (5)
O4^vi^—Na2—O4^xv^	150.7 (17)	K1—K2—O1^xviii^	85.9 (5)
Na1—Na2—O3^xvii^	74.3 (11)	K1^viii^—K2—O1^xviii^	64.7 (2)
O4^vi^—Na2—O3^xvii^	83.4 (9)	K1^xi^—K2—O1^xviii^	119.1 (6)
O4^xv^—Na2—O3^xvii^	83.4 (9)	O2—K2—O1^xviii^	69.3 (6)
Na1—Na2—Na2^xv^	180 (2)	O2^ii^—K2—O1^xviii^	119.6 (16)
O4^vi^—Na2—Na2^xv^	104.4 (9)	O1^xi^—K2—O1^xviii^	171 (2)
O4^xv^—Na2—Na2^xv^	104.4 (9)	K1^ii^—K2—O4^xx^	113 (4)
O3^xvii^—Na2—Na2^xv^	105.8 (19)	K1—K2—O4^xx^	62 (3)
Na1—Na2—O5^i^	116.3 (13)	K1^viii^—K2—O4^xx^	133.6 (15)
O4^vi^—Na2—O5^i^	131.8 (13)	K1^xi^—K2—O4^xx^	88.7 (8)
O4^xv^—Na2—O5^i^	67.9 (5)	O2—K2—O4^xx^	134.51 (15)
O3^xvii^—Na2—O5^i^	144.1 (7)	O2^ii^—K2—O4^xx^	151.78 (16)
Na2^xv^—Na2—O5^i^	63.6 (11)	O1^xi^—K2—O4^xx^	85.1 (10)
Na1—Na2—O5	116.3 (13)	O1^xviii^—K2—O4^xx^	86.7 (10)
O4^vi^—Na2—O5	67.9 (5)	K1^ii^—K2—O4^xxi^	62 (3)
O4^xv^—Na2—O5	131.8 (13)	K1—K2—O4^xxi^	113 (4)
O3^xvii^—Na2—O5	144.1 (7)	K1^viii^—K2—O4^xxi^	88.7 (8)
Na2^xv^—Na2—O5	63.6 (11)	K1^xi^—K2—O4^xxi^	133.6 (15)
O5^i^—Na2—O5	65.1 (8)	O2—K2—O4^xxi^	151.78 (16)
Na1—Na2—O5^vi^	120.9 (12)	O2^ii^—K2—O4^xxi^	134.51 (15)
O4^vi^—Na2—O5^vi^	66.1 (5)	O1^xi^—K2—O4^xxi^	86.7 (10)
O4^xv^—Na2—O5^vi^	126.3 (13)	O1^xviii^—K2—O4^xxi^	85.1 (10)
O3^xvii^—Na2—O5^vi^	58.6 (7)	O4^xx^—K2—O4^xxi^	51.3 (9)
Na2^xv^—Na2—O5^vi^	59.2 (11)	K1^ii^—K2—Na1^x^	132 (2)
O5^i^—Na2—O5^vi^	122.8 (13)	K1—K2—Na1^x^	45.0 (15)
O5—Na2—O5^vi^	89.2 (7)	K1^viii^—K2—Na1^x^	112.6 (2)
Na1—Na2—O5^xv^	120.9 (12)	K1^xi^—K2—Na1^x^	85.8 (3)
O4^vi^—Na2—O5^xv^	126.3 (13)	O2—K2—Na1^x^	87.4 (4)
O4^xv^—Na2—O5^xv^	66.1 (5)	O2^ii^—K2—Na1^x^	141.5 (14)
O3^xvii^—Na2—O5^xv^	58.6 (7)	O1^xi^—K2—Na1^x^	127.1 (11)
Na2^xv^—Na2—O5^xv^	59.2 (11)	O1^xviii^—K2—Na1^x^	48.0 (3)
O5^i^—Na2—O5^xv^	89.2 (7)	O4^xx^—K2—Na1^x^	49.6 (6)
O5—Na2—O5^xv^	122.8 (13)	O4^xxi^—K2—Na1^x^	83.8 (12)
O5^vi^—Na2—O5^xv^	62.1 (8)	K1^ii^—K2—Na1^xix^	45.0 (15)
Na1—Na2—O2^i^	56.1 (8)	K1—K2—Na1^xix^	132 (2)
O4^vi^—Na2—O2^i^	109.3 (12)	K1^viii^—K2—Na1^xix^	85.8 (3)
O4^xv^—Na2—O2^i^	57.4 (6)	K1^xi^—K2—Na1^xix^	112.6 (2)
O3^xvii^—Na2—O2^i^	121.4 (11)	O2—K2—Na1^xix^	141.5 (14)
Na2^xv^—Na2—O2^i^	123.8 (17)	O2^ii^—K2—Na1^xix^	87.4 (4)
O5^i^—Na2—O2^i^	60.3 (6)	O1^xi^—K2—Na1^xix^	48.0 (3)
O5—Na2—O2^i^	89.3 (10)	O1^xviii^—K2—Na1^xix^	127.1 (11)
O5^vi^—Na2—O2^i^	175.4 (8)	O4^xx^—K2—Na1^xix^	83.8 (12)
O5^xv^—Na2—O2^i^	122.25 (12)	O4^xxi^—K2—Na1^xix^	49.6 (6)
Na1—Na2—O2	56.1 (8)	Na1^x^—K2—Na1^xix^	130.3 (18)
O4^vi^—Na2—O2	57.4 (6)		

Symmetry codes: (i) −*x*, *y*, *z*; (ii) −*x*+1/2, *y*, −*z*+1/2; (iii) −*x*, −*y*, −*z*; (iv) *x*, −*y*, −*z*; (v) −*x*+1/2, −*y*, *z*−1/2; (vi) *x*, −*y*, −*z*+1; (vii) *x*+1/2, *y*, −*z*+1/2; (viii) *x*, −*y*+1/2, *z*+1/2; (ix) *x*, *y*, *z*−1; (x) −*x*, −*y*+1/2, *z*−1/2; (xi) −*x*+1/2, −*y*+1/2, −*z*; (xii) *x*, −*y*+1/2, *z*−1/2; (xiii) *x*−1/2, −*y*+1/2, −*z*; (xiv) −*x*+1/2, −*y*, *z*+1/2; (xv) −*x*, −*y*, −*z*+1; (xvi) *x*, *y*−1/2, −*z*+1/2; (xvii) *x*, *y*, *z*+1; (xviii) −*x*, −*y*+1/2, *z*+1/2; (xix) −*x*+1/2, −*y*+1/2, −*z*+1; (xx) *x*, *y*+1/2, −*z*+1/2; (xxi) −*x*+1/2, *y*+1/2, *z*.
